# History and Mechanism for Treatment of Intracerebral Hemorrhage with Scalp Acupuncture

**DOI:** 10.1155/2012/895032

**Published:** 2012-02-22

**Authors:** Zhe Liu, Ling Guan, Yan Wang, Cheng-Long Xie, Xian-Ming Lin, Guo-Qing Zheng

**Affiliations:** ^1^The Third Clinical College of Zhejiang Chinese Medical University, Hangzhou 310005, China; ^2^Department of Acupuncture and Moxibustion, General Hospital of the Chinese People's Liberation Army, Beijing 100853, China; ^3^Center of Neurology and Rehabilitation, The Second Affiliated Hospital of Wenzhou Medical College, Wenzhou 325027, China

## Abstract

Intracerebral hemorrhage (ICH) is an important public health problem with high rates of mortality, morbidity, and disability, but no clinically proven treatment strategy is available to date. Scalp acupuncture (SA) refers to a therapy for treating diseases by needling and stimulating the specific areas of the scalp. The evidence from clinical studies suggested that SA therapy may produce significant benefits for patients with acute ICH. However, the therapeutic mechanisms are yet not well addressed. Therefore, in this paper, we provide a comprehensive overview on the history and mechanisms of SA therapy on acute ICH. Although SA has been practiced for thousands of years in China and could date back to 5 BC, SA therapy for acute ICH develops only in the recent 30 years. The possible mechanisms associated with the therapeutic effects of SA on ICH include the influence on hematoma, brain edema, and blood brain barrier, the products released from haematoma, the immune and inflammatory reaction, focal perihemorrhagic hypoperfusion and hemorheology, neuroelectrophysiology, and so on. At last, the existence of instant effect of SA on acute ICH and its possible mechanisms are presented.

## 1. Introduction

Stroke is the second most common cause of death after heart attacks and the major cause of disability in the Western world [1], which may soon become the leading cause of death worldwide [2]. Primary intracerebral hemorrhage (ICH) is a devastating stroke with a higher death and disability rate compared to ischemic stroke and accounts for 10~15% of all strokes. However, no effective therapy to date could be established in a large trial and its outcome remains poor [3]. Furthermore, more than two-thirds of the burden of global stroke occurs in developing (low- and middle-income) countries [4]. Among developing countries, China has the largest population, about one-fifth of the world's population. Simultaneously, the incidence of hemorrhagic stroke in China was generally higher than that in the Western countries [5]. Because high proportions of ICH occur in developing countries, the setting of new research directions is sorely needed [6].

The most appreciable distinction between China and the West in treating stroke is the use of traditional Chinese medicine (TCM) which includes herbal medicine, acupuncture, and other nonmedication therapies [7, 8]. TCM has played an important role in the medical care of stroke patients for thousands of years [9]. Currently, various unconventional local therapeutic traditions are widely used for stroke treatment in China, although Level I, class A evidence of effective treatment is lacking [10]. Acupuncture therapy, which is a major therapeutic method in Chinese traditional healing systems, has also been used in stroke therapy for a long time. Scalp acupuncture (SA), also called scalp acupuncture therapy, cranial needling therapy, and scalp acupoints penetration therapy, is developed based on the traditional acupuncture science, modern anatomy, neurophysiology, and bioholographic theory. SA belongs to micropuncture system, in which filiform needle is used to penetrate specific stimulation areas on scalp. It is often applied for treatment of central nervous system disorders and various acute or chronic pains and has achieved specific curative effects [11]. Particularly, SA therapy was used in the treatment of both ischemic stroke and hemorrhagic stroke with good therapeutic effects in China [12]. Recently, a Meta-analysis done by our group showed that SA therapy could be able to improve neurological deficit in acute hypertensive ICH patients [13]. However, the mechanisms of the therapeutic effects of SA on acute ICH are not well addressed. Therefore, in this paper, we provide a comprehensive overview on the history and mechanisms of SA therapy on acute ICH.

## 2. A Brief History of Scalp Acupuncture and Its Application in Acute Intracerebral Hemorrhage

SA therapy has been practiced for thousands of years. Doctors of ancient time had already known the importance of scalp acupoints from practice, but SA develops very fast only in recent decades. A brief history of the development of SA has been summarized in Table 1. SA is originated from cranial acupuncture for disease treatment in ancient China. In the historical records-Bian Que's Biography, there may have the earliest medical record for the application of SA therapy in China. It was said that in 5 BC, Bian Que cured a patient (Guo state's crown prince) suffering from *Shi jue* (false death) with acupuncture on Baihui (GV 20) [14]. *Huangdi Neijing *(*Huangdi's Internal Classics*, HIC) established the TCM theory basis for SA therapy. In this theory, head is where all Yang-meridians meeting together and is the house of the original spirit. Head is also where convergence between meridian/channel qi and blood happens. Based on the theory “starting and terminal point of meridian/channel” and the theory “manifestation and root cause of meridian/channel”, scalp acupoints and body acupoints are closely related with extremity acupoints. Brain is the sea of marrow and is the master of Zang-fu organs and meridian/channel functional activities. Acupoints on the head can regulate function of Yin-yang, Qi-blood, and Zang-fu organs. This is the main theory basis for SA therapy. There are also many records of SA treatment cases in HIC. There are more than 50 parts in *Zhenjiujiayijing* (*A-B Classic of Acupuncture and Moxibustion*) of Jin Dynasty recording treatment of disease through head acupoints, for example, the diseases of head, face and the five sensory organs, and psychiatry and neurologic diseases, and so forth. Afterward, SA therapy gradually developed through different generations and the records were rich in medical books. Mister Cheng Danan (1899–1957) found the first professional journal of acupuncture—*Journal of Acupuncture* in October 1933. Reports are published about curing diseases through the combination of acupoints on the body and acupoints on scalp like Baihui (GV 20), Fengfu (GV 16), Shenting (GV 24), Shangxing (GV 23), Fengchi (GB 20), and Shuaigu (GB 8). But the records are rare about treating disease only with needling scalp acupoints, and also the level of curing diseases on all part of the body with scalp acupoints has not reached. Therefore, it still belonged to the traditional acupuncture system. The common prescriptions usually consisted of both scalp acupoints and distal-limb points in combination. It was mainly used for the treatment of mind illness, head, face and ear-nose-throat diseases, and so forth and did not form up an independent system.

From the 1950's, acupuncture experts began to observe the relationship between body locations of diseases with the corresponding subscalp places, and to define the associativity and effectiveness of the inspiration of micropuncture system such as auricular acupuncture, nose acupuncture, and eye acupuncture, for disease treatment. These kinds of microacupuncture points are distributed in different regions of the body as point(s) or line(s). Needling on the points of one region is able to treat problems of the other regions as well as problems of its own. The application of SA therapy increased obviously since then, and the clinical experience became richer. For example, Fang Yunpeng in Shanxi at the end of 1950's and Tang Yansong in Shanghai at the end of 1960's began to apply scalp acupuncture for curing diseases, and gradually improved this method.

In the early 1970's, SA was separated from traditional acupuncture system under the influence of neuroanatomy, neurophysiology, and bioholographic principle of modern medicine. New theories and needling methods are formed up and significant curative effects have achieved in the treatment of brain diseases. Acupuncture society put great emphasis on SA study and has formed up various scalp acupoint nomenclature systems. SA was gradually developed as an independent and complete acupuncture system. In 1971, Jiao Shunfa from Shanxi province in China first established a modern acupuncture technique combining traditional needling methods with western medical knowledge of representative areas of the cerebral cortex, including anatomy, physiology, pathology, and neurology [15]. Jiao's SA is based on the Western medical theory of nerve system and stimulates specific areas such as motor area, sensory area, and praxis area. The scalp areas corresponding to the functional locations of cerebral cortex are taken as the stimulating areas in SA for the treatment of diseases. Later, Fang Yunpeng (Shanxi) SA therapy was published in 1976 and Zhu Longyu (Shanxi) cranial acupuncture therapy published in 1979 in China [16]. The other SA therapy founders include Zhang Mingjiu (Nanjing), Tang Songyan (Shanghai), and Lin Xuejian (Shanghai) et al. Each of them proposed different diagrams and groupings of scalp acupuncture points. For example, some divided the scalp into zones or regions, while some focused on points or lines.

In 1970's, some papers were published in English journals which introduced SA therapy in the treatment of brain diseases [17–19]. In America, Dr. Zhu Minqing (Beijing) began developing his SA method in the 1970's and brought his practice to the United States and created the Chinese Scalp Acupuncture Center of USA in 1991, San Francisco. His practice now expands to Zhu's Acupuncture Medical & Neurology Center, in San Jose, CA. In 1992, he published an English-language book on his methods: *Zhu's Scalp Acupuncture *[20]. According to Dr. Zhu, *Baihui* (GV-20) is the basis for all of the scalp points. In Zhu's SA therapy, there are three main zones (designated the *Eding* zone, *Dingzhen* zone and *Dingnie* zone) subdivided into a total of 11 portions and three secondary zones, each divided into two portions (designated *Epang* 1, *Epang* 2, front zone of *Dingjie*, back zone of *Dingjie*, *Niehou* and *Nieqian*). In Japan, based on what he identified as somatotopes, Dr. Toshikatsu Yamamoto has developed what is known as Yamamoto New Scalp Acupuncture (YNSA). YNSA was presented to the world for the first time in 1973. It involves point selection through abdominal or neck test zone palpation, with extremely accurate fine needling of the appropriate scalp points [21]. In 1998, a book named *Yamamoto new SA therapy* was published [22]. Although there are many differences between Japanese and Chinese SA therapy like in the theoretical basis, the stimulating sites, and the names of acupoints, they still have many common aspects in clinical indications and manipulating methods.

As SA therapy was widely used in China and its effectiveness was verified through experience, it was gradually accepted and became a commonly used therapy in many countries around the world. The standardization of the names of acupoints was thus very necessary and urgent, so it was put on the agenda. In 1983, the World Health Organization (WHO) Western Pacific Ocean Region Committee entrusted China Acupuncture Association to prepare the scheme of Standard Nomenclature of SA lines. After the discussion in the standardization working group in 1984, 1985, and 1987, consensus of opinion has been reached and named as *A Proposed Standard International Acupuncture Nomenclature*: 3.6 Scalp acupuncture lines. This scheme was formally adopted in a science group meeting hold by WHO in 1989 and was published in 1991 [23]. This nomenclature was formulated according to the domains on the meridians and combined with the traditional penetration puncture technique (one needle penetrate two or three points). The nomenclature consists of three parts: the codes, Chinese character names, and Chinese spelling names. The codes include initials of microsystem and scalp point (MS) and the corresponding serial number. There are 14 SA point lines in all spreading in 4 regions (Table 2). The nomenclature is a great contribution to the development and popularization of SA therapy and to the international academic exchange of SA study. Acupuncture can be performed manually (manual acupuncture) or electrically (electric acupuncture), the former including penetration needling of scalp acupoints. Insert the needle subcutaneously, 0.35 mm–0.45 mm in diameter and 40 mm–70 mm in length, with an angle of 15~30°. Twirl the needle continuously or repeatedly quickly. Electra-acupuncture is applicable, but the intensity should be set appropriately. During the manipulation, blood vessels should be kept away. After plucking the needle, press the pinhole to avoid subcutaneous hematoma [24].

Therefore, SA has many schools or systerms. For example, in the SA school of Dr. Jiao, Fang et al., the theory base is mainly cerebral physiology and anatomy. The school of international standard scalp acupuncture lines is largely based on meridian theory and acupoints on the head. In a word, SA is not a single system, but a multiplicity of systems still in development.

SA therapy for acute ICH develops only in the recent 30 years (Table 3). Until 1980's, some study groups started to explore SA therapy for acute ICH. In 1991, Dong's group from Heilung-kiang University of TCM first published the clinical trial that applied scalp penetration acupuncture in the treatment of acute ICH [25] and subsequent series of studies showed that SA therapy for acute ICH were generally safe and achieved significant benefits. Based on cerebral projection on the scalp and general regulation theory, penetration needling of scalp acupoints is used in SA. Because other acupoints are also stimulated through needling one acupoint and other meridians are simultaneously stimulated through one meridian, penetration needling can synthetically modulate function of brain and body, so as to reach the goal of treatment. In 1999, a well-designed clinical trial was conducted by Li et al. [26], suggesting that SA was safe and effective in treating acute ICH. In 2006, our group published the first paper entitled “Literature foundation of scalp-acupuncture treatment of cerebral hemorrhage and its recent progress in clinical research” [27]. In 2009, our group put forward some new ways of thinking and new methodological standards on stroke study using SA, and this research may provide methodological reference for future study on the mechanism [12]. Recently, our group conducted the first Meta-analysis of SA therapy for acute hypertensive ICH [13].

## 3. Mechanisms for Treatment of ICH with Scalp Acupuncture

The main pathophysiological phases after ICH consist of direct pathological damage of hematoma information and secondary pathological damage, including early hematoma enlargement and perihematoma oedema due to the breakdown products released from the hematoma. Qureshi et al. [28] has summarized that a secondary cascade of neural injury initiated by ICH is started by products of coagulation and haemoglobin breakdown; products released by activated microglia such as oxygen free radicals, matrix metallopeptidase (MMP), complement factors, tumour necrosis factor (TNF)-*α*, interleukin (IL)-1*β*, and their expression in aquaporin (AQP)-4 astrocytes; breakdown of connective tissue in blood-brain barrier (BBB); expression of adhesion molecules that induce breakdown of the BBB, vasogenic oedema, and apoptosis in neurons and glia. The possible mechanisms for SA therapy in acute ICH were summarized in Table 4, based on the recent researches.

## 4. Influence on Hematoma, Brain Edema and Blood Brain Barrier

Early brain odema volume relative to hematoma volume makes the greatest contribution to clinical outcome [29]. In ICH patients, head CT scan showed that acupuncture on Baihui (GV 20) penetrating Taiyang (EX-HN5) improved the absorption rate of hematoma [30]. In ICH rats, acupuncture on Baihui (GV 20) penetrating Qubin (GB 7) reduced brain edema [31]. Similarly, acupuncture on Baihui (GV 20), Shuigou (GV 26), Hegu (LI 4), and Taichong (LR 3) could accelerate the extenuation of brain edema and diminish cerebral vessel permeability and brain tissue damage in acute ICH rats [32]. BBB disruption is a hallmark of ICH-induced brain injury [33]. Electric acupuncture on Baihui (GV 20) penetrating Taiyang (EX-HN5) could promote reparation of BBB damage after acute ICH in rats [34].

## 5. Influence on the Products Released from Haematoma

In recent years, more researches focused on the role of local hematoma in the formation of brain edema after ICH. The products released from the intracerebral haematoma or components of blood itself may create the material basis of brain edema. Zhang et al. [35, 36] reported that the therapeutic effects of Baihui (GV 20) penetrating Qubin (GB 7) may be through the way of inhibiting the expression of endogenous MMP-9 and AQP-4 in acute ICH rat. Thrombin plays an important role in progression of oedema and injury to nerve cells and axons surrounding the haematoma [37]. Thrombin regulates cellular functions in a large variety of cells through protease-activated receptors (PARs). Chi et al. [38] showed that acupuncture on Baihui (GV 20) penetrating Qubin (GB 7) can effectively inhibit the expression of PAR-1 in brain tissue of the acute ICH rat.

## 6. Influence on the Immune and Inflammatory Reaction

When ICH occurs, blood components including erythrocytes, leukocytes, macrophages, and plasma proteins (thrombin, plasmin, etc.) immediately enter the brain. An inflammatory cascade response follows that involves enzyme activation, mediator release, inflammatory cell migration, glial activation, brain tissue breakdown, and repair [39]. The therapy of Baihui (GV 20) penetrating Qubin (GB 7) can inhibit the expression of IL-1*β* in brain tissue surrounding the hematoma of acute ICH rat [40]. Furthermore, scalp needling led to rapid decrease of serum IL-6 content and improved nervous function in the patients of acute ICH [41]. Thirdly, acupuncture on Baihui (GV 20) penetrating Taiyang (EX-HN5) has an obvious inhibitory effect on the immune-inflammatory reaction mediated by TNF-*α* expression in acute ICH rats [42]. Fourthly, acupuncture on Baihui (GV 20) penetrating Qubin (GB 7) promoted heat shock protein 70 (HSP70) mRNA expression in brain tissue in acute ICH rats [43]. At last, acupuncture on Baihui (GV 20) penetrating Taiyang (EX-HN5) could significantly improve immune function in acute ICH patients. CD3 and CD4/CD8 level increased, while CD8 level decreased. Immune function of patient with acute ICH could benefit greatly from SA therapy [44].

## 7. Influence on Focal Perihemorrhagic Hypoperfusion and Hemorheology

Cerebral ischemia has been proposed as a mechanism contributing to secondary neuronal injury after ICH, but focal perihemorrhagic hypoperfusion is recently considered as a consequence of reduced metabolic demand, that is, diaschisis rather than a sign of ischemia [45]. Electric acupuncture on Baihui (GV 20) penetrating Taiyang (EX-HN5) improved mitochondrial energy metabolism in brain, upregulated the activity of hondriosome (Succinate dehydrogenase and Na^+^-K^+^-ATPase), and reduced the accumulation of lactate acid in the perihematoma tissue of ICH rats [46]. Scalp electroacupuncture on Baihui (GV 20) penetrating Taiyang (EX-HN5) in acute ICH rat can delay glucose transporter-1 (Glut-1) and Glut-1 mRNA expressions at brain tissue surrounding the hematoma [47]. Acupuncture on Baihui (GV 20) penetrating Taiyang (EX-HN5) lowered plasma endothelin (ET) level and neuron specific enolase (NSE) level and heightened calcitonin gene-related peptide (CGRP) level in the patient of acute ICH. These effects may regulate the function of blood vessel, reduce cerebral injury, and improve the neurological function of patients with acute ICH [48, 49].

## 8. Influence on Neuroelectrophysiology

Electrical activity of brain is a good indicator that directly reflects cerebral function state. Dong et al. [50] did a series of experimental studies on the influence and bidirectional regulation of SA on the electric activities of pain reaction neurons in acute ICH rats. The results showed that nerve cell function in local, neighboring, and remote area of ICH lesion, and the opposite side of lesion was damaged in the early stage after ICH. The functional activity decreased and the dysfunction area was far more than haematoma and its peripheral area. At the early stage SA significantly reduced these injuries after ICH. In addition, SA has bidirectional and beneficial regulation effect on electric activity of pain-reaction neurons in experiment animals. Zhao et al. [51] reported that acupuncture on Baihui (GV 20) penetrating Taiyang (EX-HN5) could recover abnormal somatosensory-evoked potentials (SEPs) and improve coordination and compensation functions among cortical functional areas, thus improve the neurological function in acute ICH patients.

## 9. Others

Acupuncture on Baihui (GV 20) penetrating Qubin (GB 7) promoted the nerve growth factor (NGF) gene expression around the hematoma and had a role in neuroprotection in acute ICH rats [52]. In addition, acupuncture on Baihui (GV 20) penetrating Qubin (GB 7) promoted the expression of endogenous glial cell-line-derived neurotrophic factor (GDNF) in acute ICH rats [53]. Acupuncture on Baihui (GV 20) penetrating Taiyang (EX-HN5) could lower the level of serum S-100B, increase the level of insulin-like growth factor 1 (IGF-1), improve the nervous functional deficit, improve the coordination and compensation function, and improve the general nervous function between cortical functional areas, so as to improve activity of daily living in acute ICH patients [54, 55].

## 10. Mechanism of Instant Effect

Instant effect means that in 10 minutes after acupuncture, muscle force in paralysis side could elevate 2 grades or more. Inoue et al. [56] showed that SA indeed has rapid and powerful effects to remove limb paralysis caused either by cerebral infarct or by cerebral haemorrhage in rats. Dong et al. [57, 58] found that 60.71% (34/56) ICH patients showed instant effect in SA group, while no case in drug-therapy and surgery haematoma aspiration group. This study showed exact instant effect of SA therapy. The mechanisms may be that SA improved the disorder of cerebral blood flow in ischemic region, or that it changed the excitability of cerebral cortex nerve cell and led to the retroconversion of the excitability of cerebral nerve cell that was depressed by the stimulation of hemorrhage or the oppression of haematoma. The generalization effect of projection capability disappeared and the nerve cell in shock or resting state after cerebral hemorrhage was awakened. Excitability of nervous cell recovered quickly and the cerebral compensation ability increased. The brain electric activity speeded up and might promote the conduction of central nervous system. This may be one of the mechanisms for the instant effect for SA. Wang et al. [59] showed that SA may regulate the electrophysiological activity of brain nerve cell and changed excitability of cerebral cortex nerve cell, thus awakened brain nerve cells that were in the state of shock or dormancy after ICH. Song et al. [60] indicated that the instant effect of SA treatment for ICH was through delaying the inhibition and death of neurons in the hematoma, relieving the inhibition of neuron discharge in the area surrounding hematoma, and reducing the influence on latency of evoked discharge. SA therapy modulated the state of neuronal function and promoted the recovery of neuronal function. Therefore, the existence of instant effect of SA illustrated the reliability of curative effect of SA on acute ICH, the importance of early treatment, and the scientific efficiency of region division and acupoint selection. The instant effect of SA, the possible mechanisms, and treatment course would be important questions for the study of SA therapy for ICH.

## 11. Summary

Recent animal and clinical studies have provided important information about the mechanisms of SA therapy for acute ICH. Current evidences indicate that various mechanisms are associated with SA therapy for ICH-induced brain injury. They are the influences on hematoma, brain edema- and BBB, the products released from haematoma, the immune and inflammatory reaction, focal perihemorrhagic hypoperfusion and hemorheology, neuroelectrophysiology, and so on. A better understanding of the role of the products released from haematoma and their effect on SA therapy could have profound implications for treatment of ICH patients. Functional magnetic resonance imaging (fMRI) will contribute to revealing the therapeutic mechanisms of SA therapy for ICH in the future, but there are few studies in this field till now. In the near future, SA therapy could offer potential novel promise in the therapeutic approach to acute ICH. However, the clarification of the mechanisms of SA therapy for ICH is urgently needed for better guidance of the ICH treatment and improvement of the prognosis of patients.

## Figures and Tables

**Table 1:** Brief History of the Development of Scalp Acupuncture.

Year	Author and/or work	Contribution
5 BC	Bian Que's biography	The earliest medical record for the application of SA therapy in China
221 BC–220 AD	*Huangdi Neijing *(*Huangdi's internal classics*)	Establishment of the TCM theory basis for SA therapy.
220 AD–1950 AD	There were rich records of SA therapy in the medical books.	SA therapy developed through different generations, but it still belonged to the traditional acupuncture system.
1950's	SA became one part of micropuncture systems.	SA began to form up an independent system.
1970's	Jiao Shunfa SA therapy, Fang Yunpeng SA therapy, Zhu Longyu SA therapy, and Zhu Minqing SA therapy, Yamamoto et al. new SA	SA therapy developed rapidly and has formed various scalp acupoint nomenclature systems.
1980's–1990's	WHO Scientific Group on International Acupuncture Nomenclature	The international standard SA lines were approved by scientific group of WHO.

SA, Scalp Acupuncture. WHO, World Health Organization.

**Table 2:** Nomenclature of scalp acupuncture lines by scientific group of WHO.

Regions	English name and location	Pinyin name	Han character name	Alphan-umeric code
Forehead region	Middle line of forehead 1 cun from GV 24 straight down along the meridian.	Ézhongxiàn	*額中* *線*	MS1
	Lateral line 1 of forehead 1 cun from BL3 straight down along the meridian.	Épángxiàn I	*額旁*1*線*	MS2
	Lateral line 2 of forehead 1 cun from GB15 straight down along the meridian.	Épángxiàn II	*額旁*2*線*	MS3
	Lateral line 3 of forehead 1 cun from the point 0.75 cun medial to ST8 straight down.	épángxiàn III	*額旁*3*線*	MS4

Parietal region	Middle line of vertex from GV 20 to GV 21 along the midline of head.	Dingzhongxiàn	*頂中* *線*	MS5
	Anterior oblique line of vertex-temporal from qiánshéncong *前神衝* (one of the four acupuncture points collectively designated as Ex-HN1, 1 cun anterior to GV 20) obliquely to GB6.	Dingniè qiánxiéxiàn	*頂顳* *前斜* *線*	MS6
	Posterior oblique line of vertex-temporal from GV 20 obliquely to GB7.	Dingniè hòuxiéxiàn	*頂顳* *後斜* *線*	MS7
	Lateral line 1 of vertex 1.5 cun lateral to middle line of vertex, 1.5 cun from BL6 backward along the meridian.	Dingpángxiàn I	*頂旁*1*線*	MS8
	Lateral line 2 of vertex 2.25 cun lateral to middle line of vertex, 1.5 cun from GB17 backward along the meridian.	Dingpángxiàn II	*頂旁*2*線*	MS9

Temporal region	Anterior temporal line from GB4 to GB6.	Nièqiánxiàn	*顳前* *線*	MS10
	Posterior temporal line from GB8 to GB7.	Nièhòuxiàn	*顳後* *線*	MS11

Occipital region	upper-middle line of occiput from GV 18 to GV 17	Zhenshàng zhèngzhongxiàn	*枕上* *正中* *線*	MS12
	Upper-lateral line of occiput 0.5 cun lateral and parallel to upper-middle line of occiput.	Zhenshàng pángxiàn	*枕上* *旁線*	MS13
	Lower-lateral line of occiput 2 cun from BL9 straight down.	Zhenxià pángxiàn	*枕下* *旁線*	MS14

**Table 3:** Brief history of SA therapy for acute intracerebral hemorrhage.

Year	Author and/or work	Contribution
1980's	Some study groups	Began to explore SA therapy for acute ICH.
1992	Dong et al. 1992 [25]	First clinical trial of the SA therapy for acute ICH.
1999	Li et al. 1999 [26]	A well-designed clinical trial to study the therapeutic effect of SA in treating acute ICH and its mechanisms.
2006	Zheng et al. 2006 [27]	A first review of this field entitled “Literature foundation of scalp-acupuncture treatment of ICH and its recent progress in clinical research”.
2009	Zheng 2009 [12]	To provide methodological reference for future mechanism study on stroke study using SA.
2010	Zheng et al. 2010 [13]	A first Meta-analysis of SA therapy for acute hypertensive ICH.

SA, Scalp Acupuncture. ICH, intracerebral hemorrhage.

**Table 4:** Possible mechanisms for treatment of acute ICH with scalp acupuncture.

Possible targets	Possible mechanisms
Influence on hematoma, brain edema and, blood brain barrier	Improve the absorption rate of hematoma, reduce brain edema, diminish cerebral vessel permeability, and promote reparation for BBB damage.
Influence on the products released from haematoma	Inhibit the expression of MMP-9, AQP-4, and PAR-1.
Influence on the immune and inflammatory reaction	Inhibit the expression of IL-1*β*, decrease serum IL-6 level, inhibit TNF-*α* expression, and promote HSP70 expression. Increase CD3 and CD4/CD8 level, while decreasing CD8 level.
Influence on focal perihemorrhagic hypoperfusion and hemorheology	improve energy metabolism, upregulate the activity of hondriosome (Succinate dehydrogenase and Na^+^-K^+^-ATPase), reduce the accumulation of lactate acid, delay Glut-1 expression, lower plasma ET level and NSE level, and heighten CGRP level.
Influence on neuroelectrophysiology	Relieve the inhibitive generalization of the whole brain neuron function, have bidirectional and beneficial regulation effect on electric activity of pain-reaction neurons, and recover abnormal SEPs.
Others	Promote the NCF gene expression, promote the expression of endogenous GDNF, lower the level of serum S-100B, and promote the level of IGF-1.

BBB, blood-brain barrier. MMP-9, matrix metallopeptidase-9. AQP-4, aquaporin-4. PAR-1, protease-activated receptor-1. IL, interleukin. TNF-*α*, tumour necrosis factor *α*. HSP70, heat shock protein 70. Glut-1, glucose transporter-1. ET, endothelin. NSE, neuron specific enolase. CGRP, calcitonin gene-related peptide. SEPs, somatosensory-evoked potentials. NCF, nerve growth factor. GDNF, glial cell line-derived neurotrophic factor. IGF-1, insulin-like growth factor 1.
